# Brain-derived neurotrophic factor measurements in mouse serum and plasma using a sensitive and specific enzyme-linked immunosorbent assay

**DOI:** 10.1038/s41598-023-34262-0

**Published:** 2023-05-12

**Authors:** Andrew Want, James E. Morgan, Yves-Alain Barde

**Affiliations:** 1 School of Optometry and Vision Sciences, Cardiff University, Cardiff, CF24 4HQ UK; 2 School of Bioscience, Cardiff University, Cardiff, CF10 3AX UK

**Keywords:** Neurotrophic factors, Assay systems, Platelets

## Abstract

This study is about the quantification and validation of BDNF levels in mouse serum and plasma using a sensitive immunoassay. While BDNF levels are readily detectable in human serum, the functional implications of these measurements are unclear as BDNF released from human blood platelets is the main contributor to the serum levels of BDNF. As mouse platelets do not contain BDNF, this confounding factor is absent in the mouse. Accordingly, BDNF levels in mouse serum and plasma were found to be indistinguishable at 9.92 ± 1.97 pg/mL for serum and 10.58 ± 2.43 pg/mL for plasma (*p* = 0.473). These levels are approximately a thousand times lower than those measured in human serum and pre-adsorption with anti-BDNF, but not with anti-NGF or anti-NT3 monoclonal antibodies, markedly reduced the BDNF signal. These results open the possibility to explore the relevance of BDNF levels as a biomarker in accessible body fluids using existing mouse models mimicking human pathological conditions.

## Introduction

The diagnosis of neurodegenerative diseases and the monitoring of therapeutic interventions would greatly benefit from the development and validation of relevant biomarkers. Given the multiple roles of brain-derived neurotrophic factor (BDNF), it would be desirable to have reliable and efficient methods to measure BDNF levels in suitable animal models and relevant body fluids. While several BDNF enzyme-linked immunosorbent assays (ELISAs) are available, they only allow measurements in tissues where BDNF levels are comparatively high such as in brain extracts or human serum. By contrast, the sensitivity of these ELISAs is not sufficient to measure BDNF levels in mouse serum^1–4^. As the mouse is the most commonly used animal model to test the relevance of biomarkers and possible correlations with conditions reflecting human diseases, we set out to measure BDNF levels in mouse blood samples using a commercially available, highly sensitive, ELISA. In mice, unlike humans, the *Bdnf* gene is not expressed at significant levels in megakaryocytes^3^, the source of BDNF stored in human platelets and released upon platelet activation and degranulation during the process of blood clotting. Nonetheless, recent results indicate that BDNF *is* present in mouse blood and originates from sources other than platelets, including the skeletal musculature^4^. Furthermore, the BDNF in mouse blood was shown in the same study to be of physiological relevance and able to activate the truncated BDNF receptor TrkB, designated TrkB.T1, expressed by pancreatic β cells^4^. As the TrkB.T1 receptor is widely expressed outside the nervous system, it appears likely that blood-derived BDNF targets tissues beyond the endocrine pancreas. The extent to which variations in BDNF levels in mouse blood correlate with conditions affecting the function of the nervous system remains unclear as it has not yet been possible to quantify BDNF levels in mouse blood.

We report the characterisation and validation of a BDNF ELISA allowing measurements of BDNF levels in mouse blood. These levels were three orders of magnitude lower than those found in primates^5^ and in line with the lack of detectable *Bdnf* gene expression in mouse megakaryocytes; no differences in BDNF levels were found between mouse serum and plasma.

## Results

**Figure 1:**
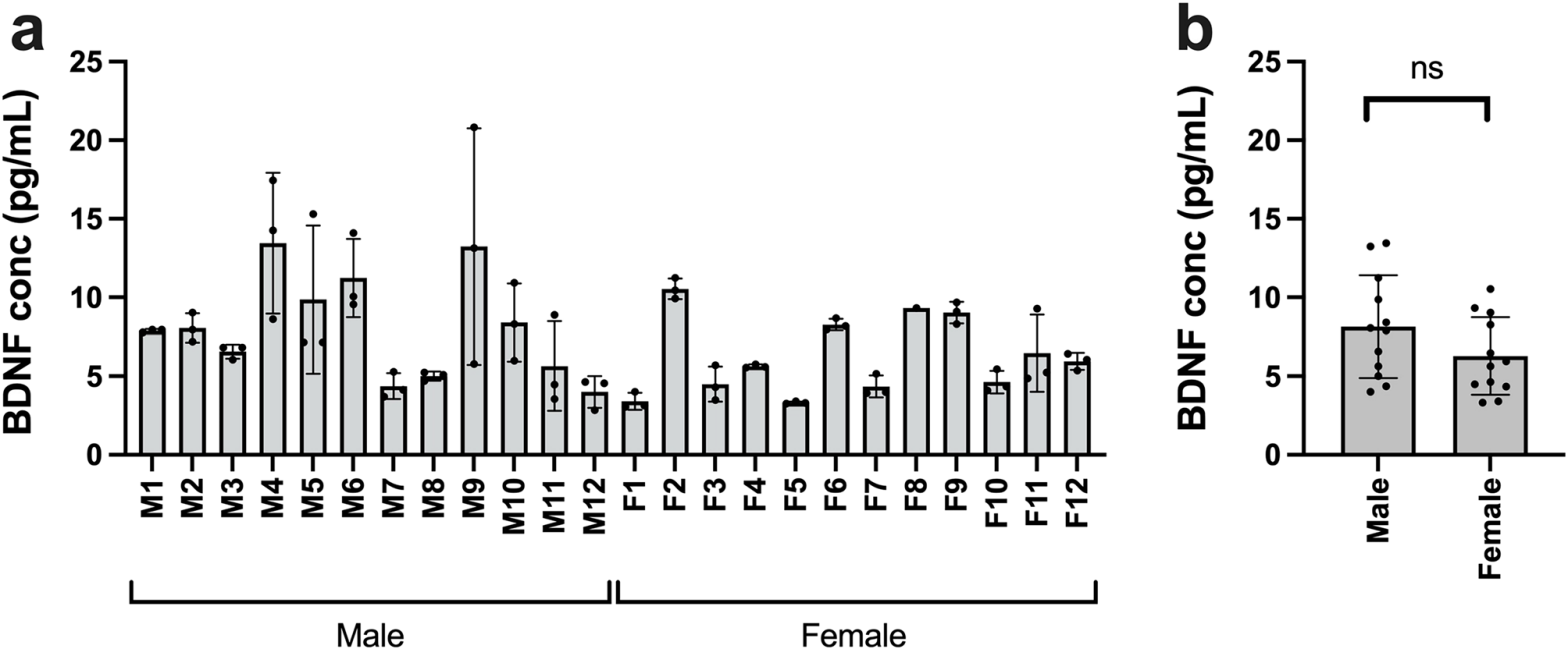
BDNF ELISA results of mouse serum samples using the mature BDNF ELISA Kit *Wako*, High Sensitive (Fujifilm, Wako #298–83,901). (**a**) BDNF concentration in pg/mL of serum samples from C57BL6/J mice. Points show concentrations for each triplicate, column shows mean of the three triplicates for samples from each individual, error bars show standard deviation of the three triplicates. (**b**) Mean serum BDNF concentration of male and female C57BL6/J mice, *p* = 0.128, unpaired *t* test. Points indicate concentration for individual animals (mean of triplicates), column indicates group mean, error bars show standard deviation.

**Figure 2:**
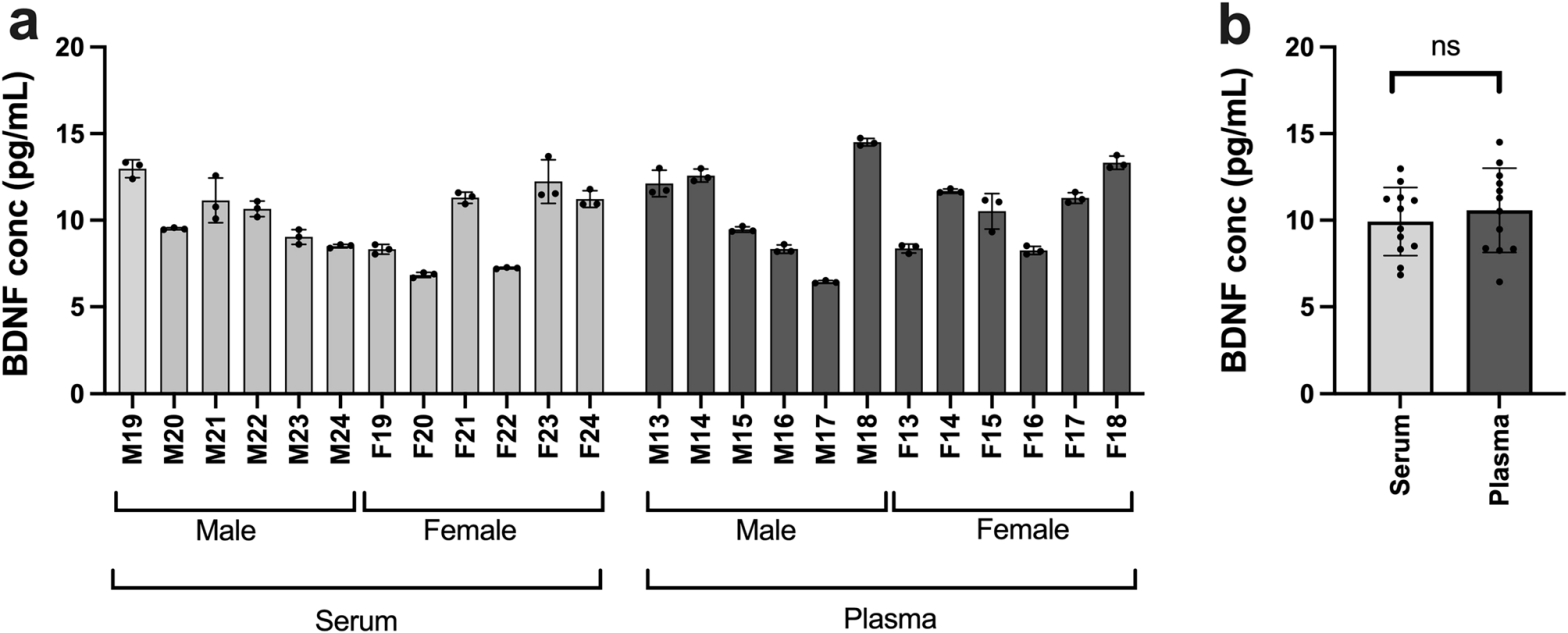
BDNF ELISA results of mouse serum and plasma samples. (**a**) BDNF concentration in pg/mL of serum and plasma samples from C57BL6/J mice. Points show concentrations for each triplicate, column shows mean of the three triplicates for samples from each individual, error bars show standard deviation of the three triplicates. (**b**) Mean concentration of serum and plasma samples. Points indicate concentration for individual animals (mean of triplicates), column indicates group mean, error bars show standard deviation, *p* = 0.473, unpaired *t* test.

Blood samples were collected from adult male and female mice and BDNF levels determined according to the manufacturer instructions (see “Methods” section). The mean BDNF concentrations in serum were found to be in the low pg/ml range and not significantly different between males and females, with *p* = 0.126, unpaired *t* test (see Fig. 1a,b). While the reported serum levels of BDNF in humans vary considerably (see for example Polacchini et al. 2015^6^ and Naegelin et al. 2018^7^ and references therein), they are approximately a thousand times higher in humans, reflecting the release of platelet content during blood coagulation. The lack of significant expression of the *Bdnf* gene in mouse megakaryocytes and the associated lack of BDNF in Western blot experiments with mouse platelet lysates^3^, prompted us to measure the levels of BDNF in plasma, both in male and female animals using the same BDNF ELISA as for serum (Fig. 2a,b). Serum samples were collected from an additional 12 mice (6 males, 6 females, all age 8 weeks) and plasma samples from a further 12 mice (6 males, 6 females, all aged 8 weeks). Mean BDNF concentrations did not significantly differ: 9.92 ± 1.97 pg/mL for serum and 10.58 ± 2.43 pg/mL for plasma (*p* = 0.473, unpaired *t* test). No difference was found between males and females in either serum or plasma: male serum 10.31 ± 1.64 pg/mL, female serum 9.53 ± 2.34 pg/mL (*p* = 0.512), male plasma 10.58 ± 3.01, female plasma 10.57 ± 1.98 pg/mL (*p* = 0.999, unpaired *t* test). The data confirm that in mice, platelets are not the source of BDNF found in blood, in contrast to humans where they contain a major reservoir (see above), resulting in serum concentrations of BDNF in humans approximately tenfold higher than in plasma^7,8^.

**Figure 3:**
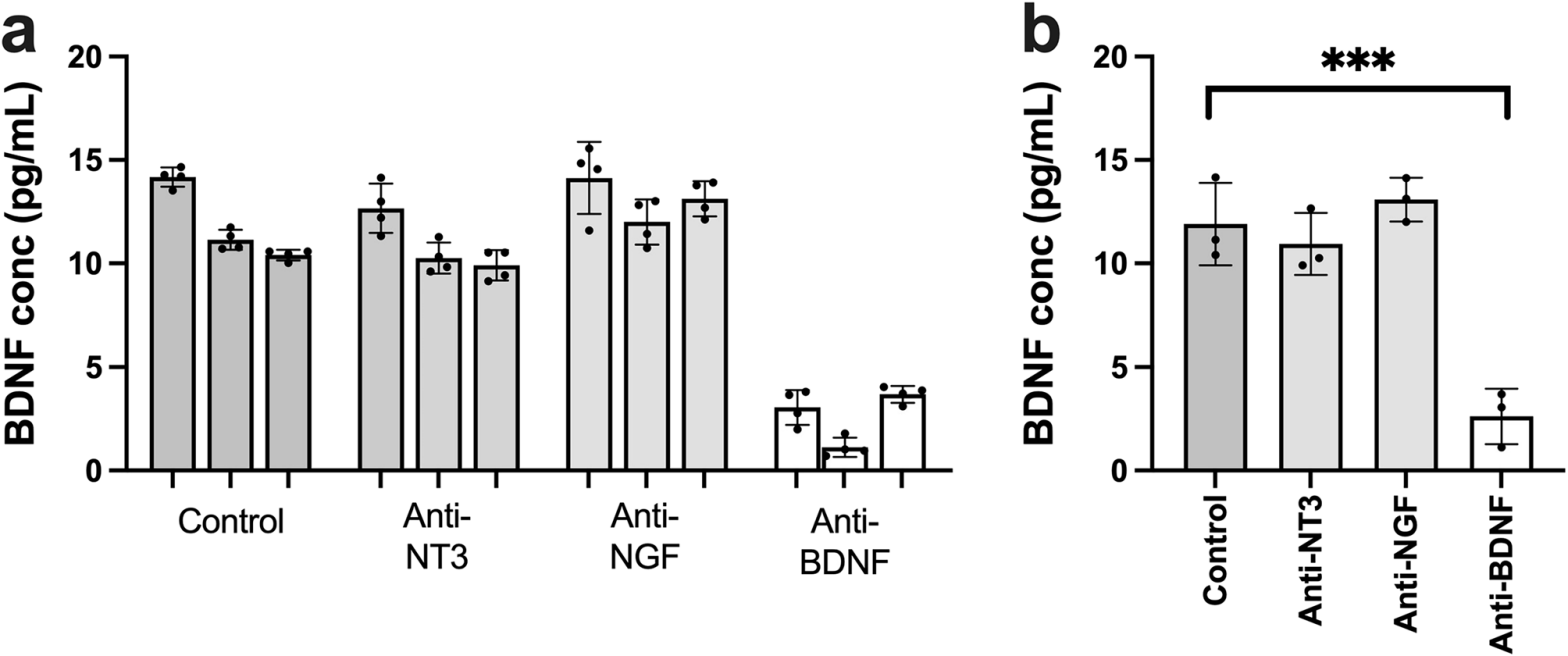
BDNF ELISA results of a pooled serum sample following incubation with antibodies to different neurotrophins. (**a**) BDNF concentration of a pooled serum sample following incubation with protein G beads alone (Control), Anti-NT3 coated protein G beads, Anti-NGF coated Protein G beads and Anti-BDNF coated protein G beads. Points show concentrations for each triplicate, column shows mean of the three triplicates for samples from each individual, error bars show standard deviation of the three triplicates. (**b**) Mean serum BDNF concentration following incubation with protein G beads alone (Control), Anti-NT3 coated protein G beads, Anti-NGF coated Protein G beads and Anti-BDNF coated protein G beads. Points indicate concentration for individual sample (mean of triplicates), column indicates group mean, error bars show standard deviation, *p* = 0.0001 one-way ANOVA.

While the BDNF ELISA values in mouse serum and plasma are significantly above background, they are very low indeed and a likely explanation for previous failures to detect BDNF in mouse serum samples. However, the low intensity of the luminescence ELISA signal raises the question as to whether this signal truly reflects the presence of BDNF in mouse serum or plasma. In an attempt to clarify this critical point, a BDNF monoclonal antibody, designated AB#9 and extensively characterised in previous studies^9,10^, was solid-phase bound to bead-coupled Protein G and used to test whether this antibody would decrease the BDNF signal following a 3 h incubation of mouse serum samples. The results indicate that such was indeed the case, with no reduction in signal seen in control serum samples incubated with Protein G beads alone (Fig. 3a,b). Since BDNF is a member of the small family of neurotrophins including nerve growth factor (NGF) and Neurotrophin-3 (NT3), we also checked that the reduction in BDNF signal was specific to BDNF binding. Protein G beads coated with anti-NGF^11^ or anti-NT3 monoclonal antibodies^12^ were then incubated with mouse serum samples and BDNF levels measured in the supernatants. In contrast to the results obtained with the BDNF monoclonal antibody AB#9, no significant differences were observed when the serum supernatant were assayed before and after adsorption with anti-NGF or anti-NT3 antibodies (Fig. 3a,b). Taken together, these results suggest that the signal detected by the BDNF ELISA used in the present study specifically reflects the presence of BDNF in mouse serum and not of NGF or NT3, even though these 3 proteins form closely related homodimers as exemplified by their similar binding affinities for the neurotrophin receptor p75^13^*.*

## Discussion

**Figure 4:**
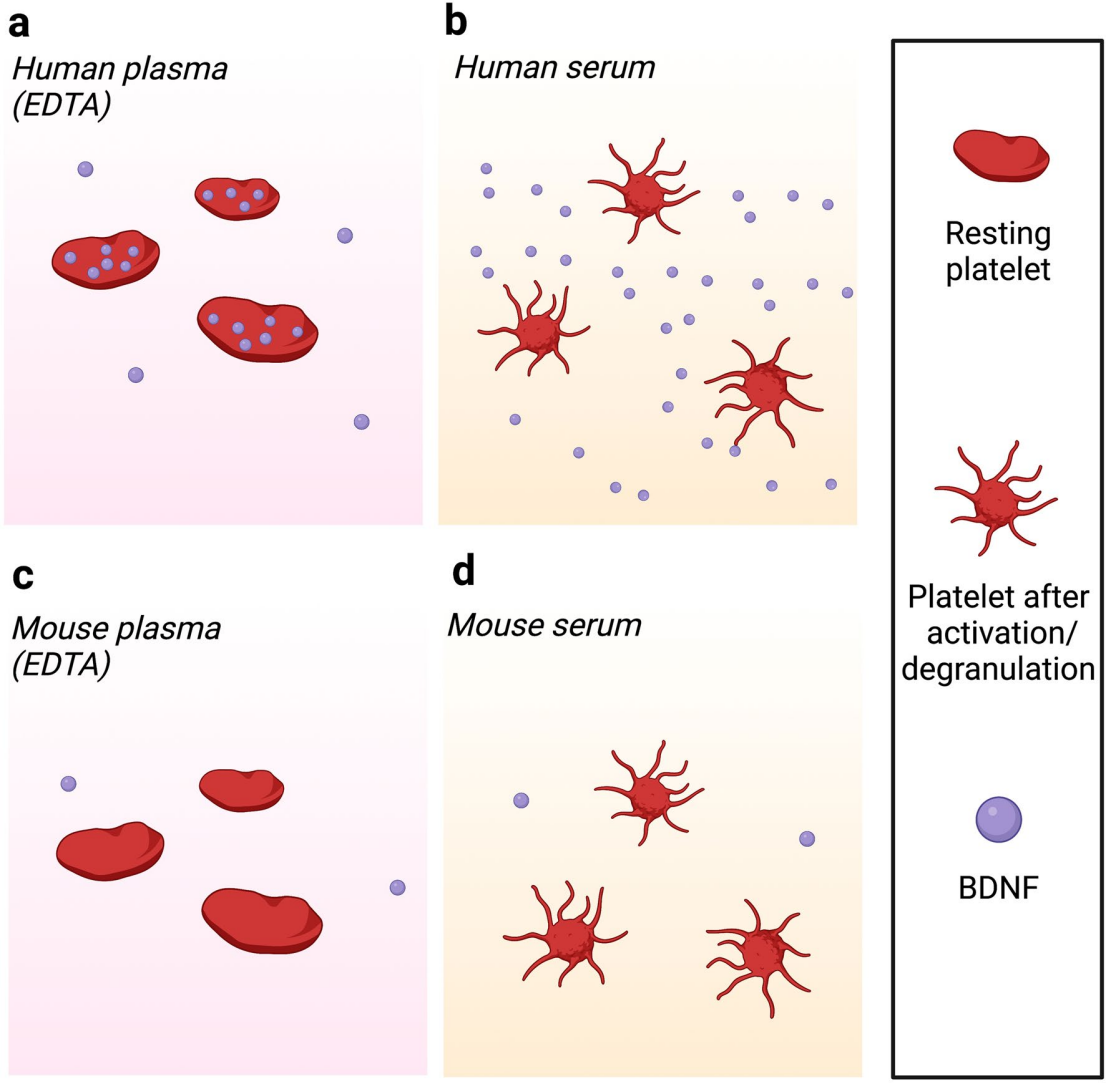
BDNF levels in mouse serum and plasma are unchanged after platelet activation/degranulation as it originates from sources such as skeletal muscle. Platelets remain in a resting state in a plasma preparation due to the addition of EDTA. For humans this means that the large reservoir of BDNF in platelets remains within the platelet granules (**a**). Serum preparation causes platelet activation and degranulation, leading to a much higher concentration of BDNF detectable in serum (**b**). Mice do not express BDNF in megakaryocytes/platelets and the concentration of BDNF was therefore unaffected by platelet activation. The similarity between plasma (**c**) and serum (**d**) concentrations suggests the small amount of BDNF is derived from a non-platelet source. Created using Biorender.com.

The main result of this study is that BDNF levels can be readily quantified in mouse blood with a commercially available ELISA validated using BDNF-specific antibodies. As expected, given previous failures to quantify BDNF levels in mouse serum by ELISAs^1,3^, these levels are remarkably low when compared with BDNF levels determined in human serum (see Introduction). This species difference can readily be explained by the lack of detectable levels of BDNF in mouse platelets, unlike the case with human platelets^3^. The similarity between the BDNF concentrations in serum and plasma further demonstrates that the BDNF detected in mouse serum is *not* derived from platelets in contrast with human samples. Indeed, plasma collection from fresh blood, as opposed to serum, includes the addition of EDTA at the time of blood withdrawal, which prevents platelet activation and degranulation (see Fig. 4 and “Methods” section). The presence, but not the quantification, of BDNF in mouse plasma has been reported in a recent study by Western blots using a BDNF enrichment procedure involving solid phase-bound TrkB reagents^4^. This same study also determined that a major source of blood BDNF in the mouse, possibly is the skeletal musculature.

We note that some mouse samples showed a wider than expected spread of the triplicate measurements (see Fig. 1a, samples M4, M5, M9) suggesting that the positions of the samples in the 96-well plate may have contributed to this variability, with one sample in a corner well and 2 others adjacent a sample with much higher luminescence. Although the 96-well plate is opaque, luminescence overspill from neighbouring wells could contribute to some of the triplicate variability observed. In practice this problem could be mitigated by avoiding placing samples anticipated to have low luminescence values next to samples at the high values of the BDNF standard curve.

As well as being highly sensitive, the BDNF ELISA kit used here is also specific for BDNF as adsorbing mouse serum samples with the BDNF monoclonal antibody AB#9 significantly decreases the BDNF ELISA signal (Fig. 3b). This decrease was not observed when AB#9 was replaced by monoclonal antibodies raised against NGF or NT3 (Fig. 3a). With regard to target specificity, it should be noted that AB#9 was raised against the BDNF native protein^9^ whereas the anti-BDNF monoclonal used as capture antibody in the ELISA kit was raised against a peptide corresponding to the amino terminal of mature BDNF. It is therefore unlikely that the two BDNF monoclonal antibodies used in this study recognise identical epitopes. Additionally, serum preparation leads to the release of a large quantity and variety of growth factors and cytokines from platelets, for example platelet-derived growth factor (PDGF) and TGF-β1^14,15^. It is therefore remarkable that no difference was seen in BDNF concentrations between the mouse plasma and serum samples, further demonstrating the specificity of the ELISA kit used in the study.

The availability of a robust method for the quantification of BDNF in mouse serum, even at very low levels, opens the possibility to now explore the role of BDNF in metabolic processes, including its role in regulating the levels of blood glucose in relevant mouse models. As suggested by Fulgenzi et al. (2020)^4^, it is likely that physical exercise may increase blood levels of BDNF as well as in the skeletal musculature,. By contrast in humans, the interpretation of measurements of BDNF levels in serum suffers from the contribution of platelet BDNF, which could obscure relevant functional contributions from other tissues. While plasma concentrations of BDNF have been measured in humans, the results are highly variable, likely reflecting the degree of platelet activation during the process of blood collection. The levels of BDNF within human platelets is such that activation of even a small proportion of platelets would be sufficient to markedly affect the BDNF concentration measured in plasma. Human plasma BDNF level determinations would need additional measurements of platelet activation markers such as platelet factor 4 (PF4) that colocalizes with BDNF in ∝-granules to assess the degree of activation.

The characteristics of the BDNF ELISA described here now allow the quantification of BDNF levels in suitable mouse body fluids. This opens the possibility that mouse models mimicking conditions in humans including depression^16^, Alzheimer’s disease^17^, autism or schizophrenia^18^ could be evaluated to determine if BDNF levels in mouse serum, plasma, cerebro-spinal fluid (CSF) or aqueous humor correlate with functional phenotypes.

## Methods

### Animals

No procedures were performed on live animals, with blood sampling being completed post-mortem. Mouse studies were approved by the Cardiff University Animal Welfare and Ethical Review Body, and all experiments were performed within the guidelines of the Home Office Animals (Scientific Procedures) Act, 1986 and Institution (Cardiff University) regulations and guidelines. Methods and results have been reported in accordance with ARRIVE Guidelines. Prior to sample collection, mice were housed in M3 cages (48 × 15 × 13 cm, 510 cm^2^ floor space) with 1–5 adult mice per cage, at a controlled room temperature of 20–24 °C, and humidity of 55% ± 10%. They were maintained on a 12-h light/dark cycle with access to food ad libitum.

### Blood collection

C57BL/6 J mice (Charles River) were culled using CO_2_ and blood collected immediately after death by cardiac puncture and processed for either plasma or serum. Approximately 800–1000 µL of whole blood was collected from each mouse.

### Serum collection

Blood samples were transferred to an Eppendorf tube and left to rest at room temperature for 1 h, followed by 1 h at 4 °C. Samples were then centrifuged at 2000 G for 10 min at 4 °C. The serum was collected (from the top of the tube) and centrifuged again at 2000G for 10 min at 4 °C to clear out any remaining cells. Serum samples were then stored at − 80 °C until needed.

### Plasma collection

Blood was collected into a syringe containing 20 µL of 500 mM EDTA and gently mixed well. The samples were then centrifuged at 2000 G for 10 min at 4 °C. The top 2/3 of plasma was then carefully removed and transferred into a new tube before being recentrifuged again at 2000 G for 10 min at 4 °C for further purification. This plasma was then collected, taking care not to disturb any remaining debris in the base of the tube, and aliquoted for storage at − 80 °C until needed.

### ELISA procedure

Quantification of the BDNF concentration in serum and plasma was performed using the Mature BDNF ELISA Kit *Wako*, High Sensitive (Fujifilm, Wako #298–83,901) as per manufacturer’s instructions, using the reagents included in the ELISA kit. Briefly, samples were diluted fourfold with the kit buffer solution and the BDNF standard prepared using the reagents provided to generate a stock solution of 10 ng/mL. This stock was then mixed with the buffer provided to generate a series of standard solutions as per kit instructions. The solution initially filling the ELISA plate was then discarded and the wells washed 4 times with the Wash Solution (1X) included in the kit. The plate was inverted after each wash and blotted against clean paper towels to remove any excess liquid retained in the wells. 50 µL of diluted standard solution and of diluted samples were added to respective wells, with triplicate wells used for each standard and sample. The plate was then covered and agitated on a microplate shaker for 2 h at room temperature (20–25 °C). Following a 2-h incubation, the solution was discarded and the wells washed again 4 times with Wash Solution as above. 50 µL of Biotin-conjugated antibody solution was added to each well, the plate covered and incubated on a shaker again for 1 h at room temperature. The solution was then discarded and wells were washed 4 times with Wash Buffer as above. 50 µL of Peroxidase-conjugated Streptavidin Solution was added to each well, the plate covered and incubated on a shaker for 30 min at room temperature. After a final series of 4 washes with the Wash Buffer, 50 µL of mixed luminescent reagents 1 and 2 (1:1) were added to each well, the plate placed on a shaker for 1 min and the luminescence then measured using a 96-well microplate reader (FLUOstar Omega, BMG Labtech). Measurement was performed 10 min after the addition of the luminescent reagent. The standard solutions were used to generate a standard curve converting luminescence to BDNF concentrations used to determine the BDNF concentration in the experimental samples.

### Mouse serum incubation with protein G-bound antibodies

60 µL of Sepharose Protein G beads were washed and resuspended in PBS. They were then incubated with 30 µg of either anti-BDNF (Ab#9), anti-NGF, anti-NT3 monoclonal antibodies (see “Results” section for references) or PBS alone for 1 h on ice with intermittent vortexing. The beads were then centrifuged for 30 s at 12,000G, after which the supernatant was discarded and the beads resuspended in a further 100 mL of PBS. This was repeated three times to ensure that any remaining, unbound antibody had been removed.

Serum from a pooled sample was diluted fourfold using the buffer solution contained in the Mature BDNF ELISA Kit *Wako*, High Sensitive (Fujifilm, Wako #298–83,901). Each bead-containing solution was then resuspended in 600 µL of the diluted serum and incubated on ice for 3 h with intermittent vortexing. Samples were then centrifuged at 12,000G for 30 s and the supernatant then transferred to a new Eppendorf tube. This process was repeated three times to ensure that all Protein G beads had been removed from the serum sample. BDNF concentration was then measured using the ELISA procedure detailed in the above.

### Statistical analysis

The sample size was selected using a power calculation (Gpower)^19^ to detect a 1.25-fold difference between serum and plasma, assuming a significance level of 0.05 and with 90% power (in humans the concentration of BDNF in serum is approximately tenfold higher than in plasma). Statistical analysis was performed using GraphPad Prism software (version 8). The normality of the data sets was tested using Shapiro–Wilk test (passed normality test when alpha = 0.05). Comparison of normally distributed groups was then performed using an unpaired *t* test, or using a one-way ANOVA to compare multiple groups.

## Supplementary Information

Supplementary Information.

## Data Availability

All datasets for the raw luminescence ELISA measurements included in this study can be found in the Supplementary Information.
